# Dietary Patterns, Eating Behavior, and Nutrient Intakes of Spanish Preschool Children with Autism Spectrum Disorders

**DOI:** 10.3390/nu13103551

**Published:** 2021-10-10

**Authors:** Julio Plaza-Diaz, Katherine Flores-Rojas, María José de la Torre-Aguilar, Antonio Rafael Gomez-Fernández, Pilar Martín-Borreguero, Juan Luis Perez-Navero, Angel Gil, Mercedes Gil-Campos

**Affiliations:** 1 Department of Biochemistry and Molecular Biology II, School of Pharmacy, University of Granada, 18071 Granada, Spain; jrplaza@ugr.es (J.P.-D.); agil@ugr.es (A.G.); 2 Instituto de Investigación Biosanitaria IBS.GRANADA, Complejo Hospitalario Universitario de Granada, 18014 Granada, Spain; 3 Children’s Hospital of Eastern Ontario Research Institute, Ottawa, ON K1H 8L1, Canada; 4 Pediatric Research and Metabolism Unit, Maimónides Institute for Biomedical Research of Córdoba (IMIBIC), Reina Sofia University Hospital, University of Córdoba, Av. Menéndez Pidal, s/n, 14010 Córdoba, Spain; katherine1.flores@gmail.com (K.F.-R.); antoniogofedez@hotmail.com (A.R.G.-F.); juanpereznavero@hotmail.com (J.L.P.-N.); mercedes_gil_campos@yahoo.es (M.G.-C.); 5 CIBEROBN (Physiopathology of Obesity and Nutrition), Instituto de Salud Carlos III (ISCIII), 28029 Madrid, Spain; 6 Department of Child and Adolescent Clinical Psychiatry and Psychology, Maimónides Institute for Biomedical Research of Córdoba (IMIBIC), Reina Sofia University Hospital, Av. Menéndez Pidal, s/n, 14010 Córdoba, Spain; pmartin.psicologa@gmail.com; 7 Centre for Biomedical Research on Rare Diseases (CIBERER), ISCIII, 28029 Madrid, Spain; 8 Biomedical Research Center, Institute of Nutrition and Food Technology “José Mataix”, University of Granada, Parque Tecnológico de la Salud, Avenida del Conocimiento, s/n, 18016 Granada, Spain

**Keywords:** autism spectrum disorders, disabled children, food and nutrition, feeding behavior

## Abstract

Eating behavior problems are characteristic of children with autism spectrum disorders (ASD) with a highly restricted range of food choices, which may pose an associated risk of nutritional problems. Hence, detailed knowledge of the dietary patterns (DPs) and nutrient intakes of ASD patients is necessary to carry out intervention strategies if required. The present study aimed to determine the DPs and macro-and micronutrient intakes in a sample of Spanish preschool children with ASD compared to typically developing control children. Fifty-four children with ASD (two to six years of age) diagnosed with ASD according to the Diagnostic Manual-5 criteria), and a control group of 57 typically developing children of similar ages were recruited. A validated food frequency questionnaire was used, and the intake of energy and nutrients was estimated through three non-consecutive 24-h dietary registrations. DPs were assessed using principal component analysis and hierarchical clustering analysis. Children with ASD exhibited a DP characterized by high energy and fat intakes and a low intake of vegetables and fruits. Likewise, meat intake of any type, both lean and fatty, was associated with higher consumption of fish and dietary fat. Furthermore, the increased consumption of dairy products was associated with increased consumption of cereals and pasta. In addition, they had frequent consumption of manufactured products with poor nutritional quality, e.g., beverages, sweets, snacks and bakery products. The percentages of children with ASD complying with the adequacy of nutrient intakes were higher for energy, saturated fat, calcium, and vitamin C, and lower for iron, iodine, and vitamins of group B when compared with control children. In conclusion, this study emphasizes the need to assess the DPs and nutrient intakes of children with ASD to correct their alterations and discard some potential nutritional diseases.

## 1. Introduction

Autism Spectrum Disorders (ASD) are neurodevelopmental disorders characterized by disturbances in communication and social interaction and by the presence of restricted, repetitive patterns of behaviors, activities, and interests. These alterations are present from early childhood, although some difficulties may not manifest until the environment’s demands exceed the child’s capacity [1]. Most studies coincide with a significant increase in the prevalence of ASD, which does not seem to be explained by improving the detection systems. For 2016, ASD prevalence in the USA was 18.5 per 1000 (one in 54) children aged eight years, and ASD was 4.3 times as prevalent among boys as among girls [2]. It must therefore be considered a serious public health problem.

Children with ASD frequently have significant eating difficulties with a highly restricted range of food choices [3], and there is consensus that children with ASD have selective dietary patterns (DPs), food neophobia and sensory issues [4]. Indeed, the Diagnostic Manual-5 (DSM-5) now includes sensory symptoms in the diagnostic criteria for ASD, such as food selectivity [1]. Eating behavior problems are characteristics of ASD [5,6,7]. The dietary complications are more common among children with ASD than among the population during the first year of life from the time of introducing the complementary foods [8]. The rejection of solid foods is very frequent, and the introduction of foods with new textures, consistencies, and flavors tends to be difficult, so they preferentially consume the same foods in a repetitive manner [9,10]. Those behaviours do not seem to influence their growth long-term [4]. Still, they generate a great deal of family anxiety, becoming one of the main concerns of caregivers and family members [11,12]. Although these behaviours usually improve with time [13], the possible nutritional repercussions and the important social limitations are relevant and sensory goals should be included in treatment objectives for children with ASD [11].

The origin of eating behavior alterations is not entirely clear. Among some of the theories that can explain this phenomenon, the following stand out: (1) cognitive/behavioral alterations [14,15,16]; (2) sensory alterations [4,16,17]; and (3) gastrointestinal disturbances [18,19]. Eating problems in children and adolescents with ASD represent a concern for parents and caregivers and a potential cause of health issues. However, studies are diverse, presenting differences in the methodological steps, so their outcomes are hard to compare [20]. Some investigations have reviewed the consumption of children with ASD compared to those with typical development and determined some nutritional preferences of children with ASD. In general, they found stronger preferences for energy-dense foods like snacks, sweets, sugary beverages and juice in children with ASD. In contrast, children with ASD tend to eat less from food groups like vegetables, fruit and dairy products than children with typical development [21].

Without curative treatments and with these eating behavior alterations [22], the use of nutritional supplements and alternative medicine, which is widely used among families of ASD patients, has been greatly encouraged [23,24] when seeking clinical improvement. However, the use of nutritional supplements e.g., those containing omega-3 fatty acids [25], multivitamins, as well as a gluten-free casein-free diet [26], is not supported by the current scientific evidence, and it should not be generally recommended. 

It has been suggested that the identification and development of nutritional assessment indicators that serve as early warning signs during routine practice are of interest [4]. Indeed, the present study aimed to determine the feeding behavior, DPs, and macro-and micronutrient intakes in a sample of Spanish preschool children with ASD compared to typically developing control children of the same age.

## 2. Material and Methods

### 2.1. Study Design and Subjects

The present work is part of an observational and case-control study described elsewhere [27]. ASD patients from two to six years old in a specialized Unit for ASD in a third level hospital (Reina Sofia University Hospital, Cordoba, Spain) and diagnosed according to DSM-5 criteria and validated by the Autism Diagnostic Observation Schedule (ADOS) score were recruited. A group of healthy children of similar age to that of the ASD group that came to the hospital for minimal surgery interventions with a normal detailed clinical history, general examination, anthropometric assessment, and control analysis to rule out any pathology was used as a control group. Both groups lived in the same urban area of Córdoba (Spain), with families in similar economic and sociocultural situations and close environmental exposure. Exclusion criteria were receiving medication for ASD comorbidities and any other pathology, be receiving or have received any nutritional supplement in the last 12 months; undertaking specific diets for therapeutic purposes in the previous 12 months e.g., celiac disease; and having a percentile higher than p97 or lower than p3 in the recommended tables of anthropometry of the Spanish child population [28,29].

The diagnosis of ASD was assessed following the clinical criteria of the DSM-5 and confirmed with the completion of the ADOS. An initial sample of 55 children with ASD (46 males and 9 females) between two and six years of age was recruited, following the inclusion criteria. During the recruitment phase, a male was excluded because he was diagnosed with celiac disease (*n* = 54). Fifty-seven healthy children, after normal results as noted above, were also included in the study as a control group.

The present study was approved by the Clinical Research and Bioethics Committee of the Hospital Reina Sofía, Cordoba, Spain. It was conducted in full compliance with the fundamental principles established in the Declaration of Helsinki. The data relating to the ASD patients were collected at the time they were recruited into the study. The recruited subjects were incorporated into the study after all the criteria for inclusion were fulfilled, and informed written consent was obtained from the children’s legal guardians.

### 2.2. Variables and Data Collection

In the recruited patients diagnosed with ASD and in the control subjects, a detailed history and general physical examination was performed.

A previously modified, adapted and validated food frequency questionnaire (FFQ) with the portion sizes and food groups usually consumed by the Spanish child population was used [30]. The adequacy of food consumption was evaluated using the Nutritional Objectives of the Consensus Document of the Spanish Community Nutrition Society; these questionnaires included one item about the dietary type and amount of supplementation [31]. 

The intake of energy and nutrients was estimated from the data obtained through three non-consecutive 24-h dietary registrations (24-h-DR), registered by parents, including two weekdays and one weekend day, following the Guidance on the EU Menu Methodology of the European Food Safety Agency (EFSA) [32]. Mean daily intakes of energy and nutrients were calculated by using the computer software of the Center of Endocrinology and Clinical Research, University of Valladolid, Institute of Studies on Health Sciences of Castilla and Leon, Spain (IENVA) (https://calcdieta.ienva.org/, accessed on 1 September 2021).

### 2.3. Statistical Analysis

#### 2.3.1. General Analyses

The sample size for the study was calculated assuming a 30% difference in the mean for one of the main study variables (plasma level of docosahexaenoic acid) between children with ASD and healthy children, an α error = 0.05, a power of 0.90 (β error of 0.1) and 5% of dropout during the follow-up of the study. For these calculations, data from recent studies were used, considering that the percentages expected would be similar to several studies carried out in populations with similar characteristics to those of children with ASD. Data were expressed as mean ± standard deviation or median plus interquartile range, depending on whether each variable’s values followed a normal distribution or not. The Shapiro-Wilk normality test was used to determine the normality of variables. The comparison between the groups was carried out using the Student’s *t*-test for continuous variables when the distributions were normal and the Mann-Whitney U test when the distributions did not follow the normality. The differences between the frequencies of the sexes were studied by means of the Chi-square test. For the statistical analysis of the data, the computer program IBM SPSS 25.0 (IBM Corp., Armonk, NY, USA) was used.

#### 2.3.2. Dietary Patterns

A principal component analysis (PCA) was accomplished to identify underlying DPs using each individual’s serving average from nine food groups as input variables [31]. These food groups considered in this study were as follows: “milk and dairy products (e.g., milk, yogurt, cheese, and milkshakes)”, “cereals and pasta (e.g., bread, cereal bars, and rice)”, “fatty meat and derivates”, “fats”, “snacks, sweets, bakery and pastry (e.g., chocolate, cookies, ice cream, candies, and bakery products)”, “fruits and vegetables”, “beverages”, “fish and shellfish (e.g., white fish, bluefish, and shellfish”, and “lean meat and eggs”. This mathematical model calculates new variables (principal components) that account for the variability in the food group’s data and enables the study of covariances or correlations between variables. We interpreted only components with eigenvalues over one and factor loadings with an absolute value higher than 0.4 (which explains around 16% of variance) as the significance of factor loading depends on the sample size. A high factor score for a given pattern indicated a high consumption of the foods constituting that food factor, and a low score indicated a low intake of those foods.

The Kaiser–Meyer–Olkin (KMO) and Bartlett test of sphericity were applied to assess the sampling adequacy. KMO values >0.50 were considered. Communalities were estimated using the squared multiple correlations of each variable with all others. We retained variables with communalities higher than 0.5. Factors were orthogonally rotated (the Varimax option) to maximize the dispersion of loading within factors, facilitating interpretability. Radar maps were used to display data in the form of a two-dimensional map of nine food groups represented on axes starting from the same point.

#### 2.3.3. Dietary Patterns with Clustering Analysis

A two-step cluster analysis procedure was conducted for the automatic selection of the best number of clusters that would otherwise not be apparent on the FFQ variables. Three clusters were assumed to be the optimum number. Later, unsupervised hierarchical clustering analysis was applied to construct clusters of subjects with similar characteristics using the “pheatmap” R software package. The distance matrix was defined by Euclidean distances, and Ward’s method was used as linkage criteria to group the clusters. The agglomerative coefficient, calculated by the Agnes function, was always higher than 0.85.

Heat maps were used to visualize hierarchical clustering, which allowed us to simultaneously picture clusters of subjects and features. Hierarchical clustering was done of both the rows and the columns of the data matrix, which were re-ordered according to the hierarchical clustering result, putting similar observations close to each other. The blocks of ‘high’ and ‘low’ values are adjacent in the data matrix. A red-blue color scheme was applied for the visualization to help to find the variables that appear to be characteristic for each subject cluster.

## 3. Results

### 3.1. Subjects Characteristics

Table 1 shows the control group’s demographic characteristics, comprising 57 healthy children and the ASD group, including 54 patients. The proportion of boys with ASD was higher than that of girls. There were no significant differences in weight, height, and body mass index (BMI) between the two groups.

### 3.2. Food Group Consumption According to Food Frequency Questionnaires and Adequacy to the Spanish Society of Community Nutrition (SENC) Dietary Guidelines

Children with ASD consumed more cereals and pasta, and milk and dairy products than the control group; in contrast, ASD children consumed fewer lean meat, eggs, and beverages. The questionnaires highlighted the consumption, in shredded form, preferably of chicken or beef with vegetables, and to a lesser extent fatty meats and fish (Table 2).

Table 3 shows the food group consumption according to food frequency questionnaires and adherence to the Spanish Society of Community Nutrition (SENC) dietary guidelines for preschool children with ASD compared with the control group. A higher percentage of ASD children consumed four to six servings per day of cereals and pasta compared with the control group. A significantly higher percentage of ASD children consumed more than six servings of milk and dairy products compared with the control children. A high percentage of ASD children consumed less than three servings/wk of lean meats and eggs compared to the control children. 

In contrast, both groups of children consumed an excess of fatty meats and their foodstuffs. The majority of ASD children consumed 2–3 servings of fat daily, whereas 22.8% of the control children consumed more than 3 servings/d. Finally, more than 85% of both ASD and control children consumed more servings than those recommended for beverages, snacks, sweets, bakery, and pastry.

### 3.3. Dietary Patterns

**Figure 1:**
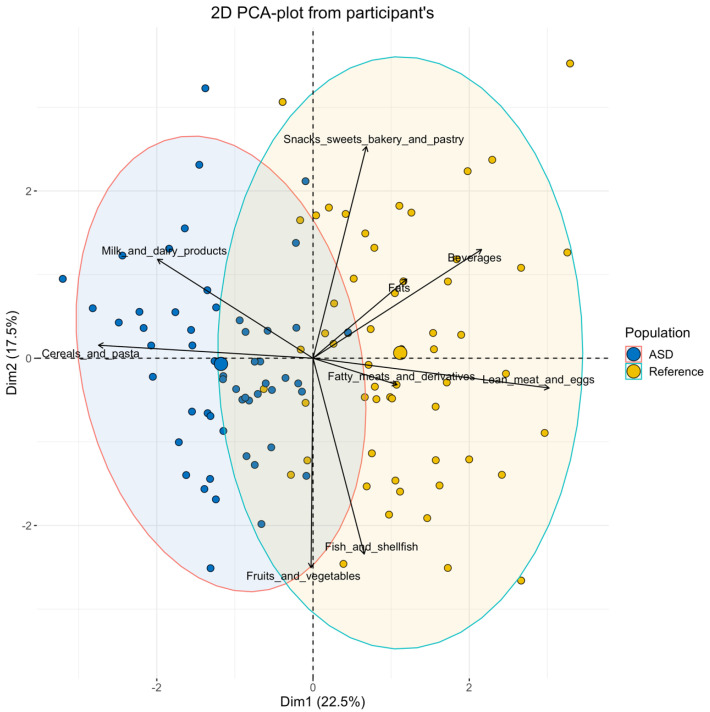
Principal component analysis (PCA) plot in the control and the children with autism spectrum disorders (ASD) according to the intake of major food groups determined by a standardized food frequency questionnaire.

Figure 1 depicts the DPs of ASD and control children evaluated through PCA’s bi-dimensional plots related to the major food groups consumed. The two dimensions, Dim1 (X-axis) and Dim2 (Y-axis) explained 22.5% and 17.5% of the total variance, respectively. Most ASD subjects separated well from the control subjects because of the increased consumption of cereals and pasta and milk and dairy products and the relatively lower consumption of lean meat and egg, fatty meats and derivatives, fish and shellfish, beverages, snacks, sweets, and bakery and pastry, compared with the control children.

A three-dimensional PCA was used to maximize the diet’s predominant food groups’ information. The combination of food group variables with the greatest amount of variability is the first principal component. The following components (second and third principal components) describe the maximum amount of remaining variability.

**Figure 2:**
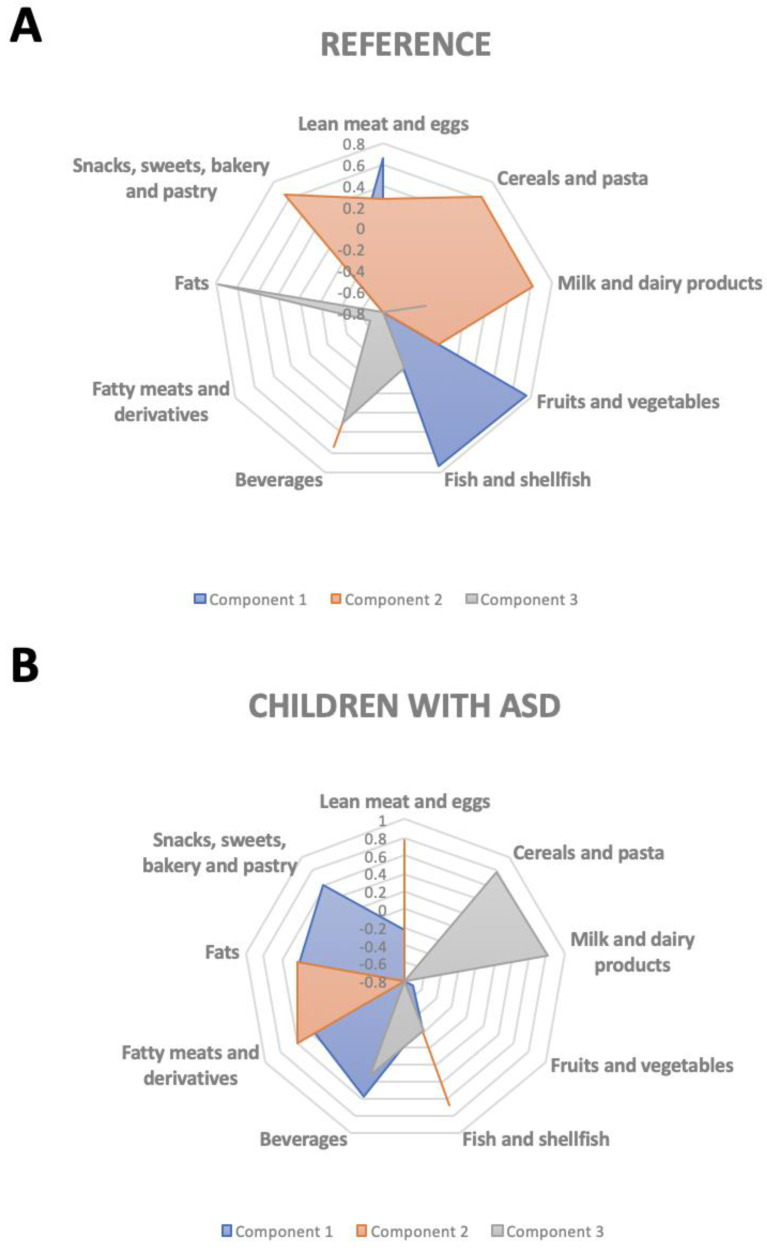
Dietary patterns extracted from the three-dimension principal component analysis of 9 major food groups. (**A**) Control group (*n* = 57), (**B**) Children with ASD (*n* = 54).

Figure 2 shows the DPs extracted from the three-dimensional PCA of nine major food groups for the control group (Panel A) and children with ASD (Panel B). In the control children, the first component was associated with a healthy pattern that included fruit and vegetables, fish, and lean meats and eggs. The second component was related to the consumption of milk and dairy, cereals and pasta to snacks, sweets, bakery, and pastry and beverages. The third component associated a high consumption of fat with low consumption of fatty meats and their derivatives. In contrast, the ASD group’s first component showed an unhealthy pattern characterized by a high relationship between low consumption of fruits and vegetables and increased consumption of snacks, sweets, bakery, and pastry, and beverages. The second component was described by the associated consumption of different meats, fish, and fat types. The third component included the related consumption of milk and dairy products and cereals and pasta.

**Figure 3:**
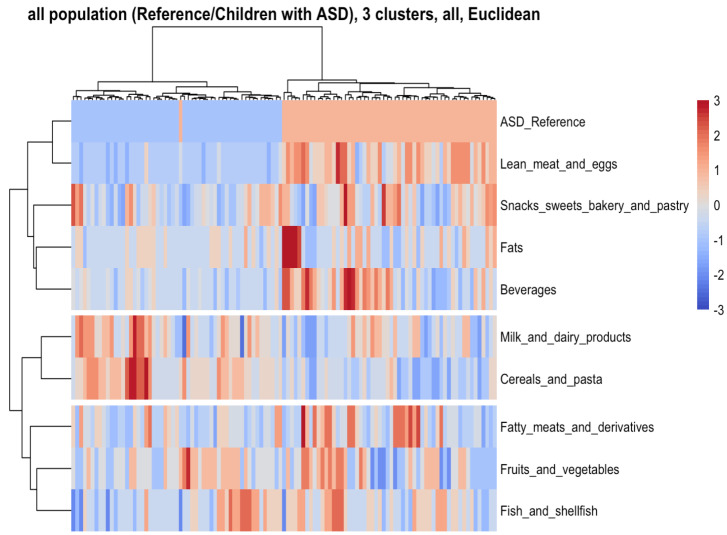
Clusters of subjects and dietary and lifestyle variables were identified via hierarchical clustering in control and ASD groups. The clusters are visually separated by longitudinal marks on vertical and horizontal faces (clusters of subjects and dietary variables, respectively). The vertical and horizontal dendrograms denote the relationship between the clusters, i.e., similar observations. The color bar refers to levels above (red) or below (blue) the mean intake of the dietary variable or means scores of food groups. Increased color intensities indicate larger differences around the mean. For the ASD control variable, ASD children are represented by the color blue and children of the control group by the color red.

Clusters of subjects classified based on their dietary characteristics are shown in a heat-map (Figure 3). There were two main clusters of children (columns) and three main dietary variables (rows) identified by the clustering algorithm. All ASD children, excepting one subject, and all healthy children, were classified into two distinct clusters (blue for the ASD and red for the control population in Figure 3. Within the first food cluster, ASD children exhibited low consumption of lean meat and eggs, snacks, sweets, bakery, and pastry, fats, and beverages compared with the control population’s children. In contrast, ASD children showed higher milk and dairy consumption and cereals and pasta for the second food cluster. In the third food cluster, the consumption of fatty meats and their derivatives, fruits and vegetables, and fish varied for different subjects in the ASD and control children.

### 3.4. Eating Behavior

**Figure 4:**
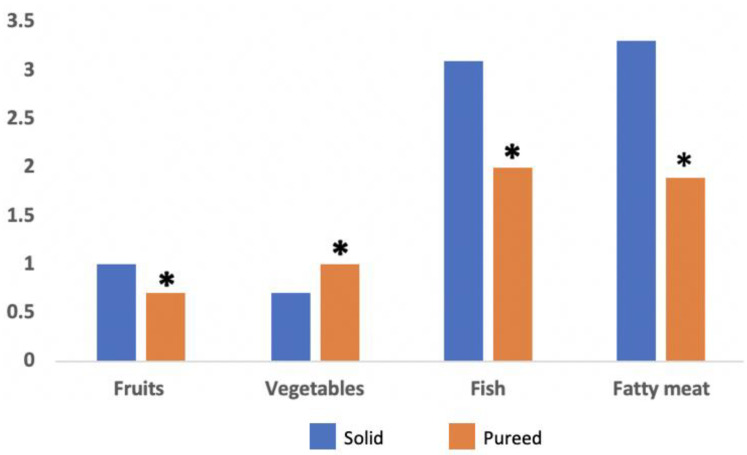
The average consumption of solids and pureed foods in Spanish preschool children with autism spectrum disorders for selected food groups (vegetables and fruit are expressed in servings/day, and fish and meat in servings/week). * *p* < 0.05.

Of the ASD 54 patients, 42% tolerated solid foods, whereas 58% tolerated only pureed foods. When comparing the number of servings for the different food groups within the ASD patient groups (solid vs. pureed), significant differences were obtained in vegetables, fruit, fish, and fatty meats (*p* < 0.05) (Figure 4). All control children tolerated solid foods.

### 3.5. Adequacy of Nutrient Intakes to the European Food Safety Authority (EFSA) Recommendations

To assess nutrient adequacy, individual usual intake was compared to the current recommendations of adequate intake (AI) and reference intake range (RI) defined by EFSA [30]. Table 4 shows the percentages of children meeting and not meeting the EFSA recommendations for energy, macronutrients, minerals, and vitamins.

The majority of ASD children had an adequate energy intake, while about 23% of the control group did not reach the EFSA recommendations. Both ASD and the control group had a higher intake of protein than recommended by EFSA. The intake of carbohydrates was similar in the two groups of children, with more than 70% following the recommendations. Nevertheless, the intake of sugars in both groups was higher than recommended in the majority of children. In contrast, the recommended intake of fiber was not reached by about 51% of ASD children compared with only 23.4% in the healthy control group, although only a statistical trend was found (*p* = 0.085). Besides, the percentage of ASD children who had an intake of saturated fat above the guidelines was higher compared with that of the control children. The intake of total polyunsaturated fatty acids was similar in both groups of children. Even the intakes of essential fatty acids [linoleic acid (LA) and α-linolenic acid (LNA)] were similar; the average energy intake was lower than that recommended by the EFSA (LA: 1.25% and 1.13%; LNA 0.13% and 0.13%, respectively). The average intake of EPA + DHA was about 0.3 g/d for the control and 0.15 g/d for ASD children, which is close to the EFSA guidelines.

About 79% of ASD children had an adequate calcium intake compared with only about 50% in the control group. Adequacies of phosphorus, magnesium, and potassium intake were similar in both groups. However, most of the subjects in both groups did not reach the AI for magnesium. Similarly, the iron, zinc, copper, and manganese intakes were lower than the corresponding AI in most ASD and in the control group. The adequacy of intake was lower in ASD children than in the control group; in fact, 100.0% of ASD children did not meet the AI for iodine (Table 4).

Regarding the vitamins, the usual individual intakes of vitamins D, E and B9 were below the recommended AI in most of ASD and control children. The intakes of vitamin A and K were similar in both groups, with about one half following the recommendations. Finally, we observed significantly higher percentages of ASD children that did not meet the EFSA guidelines for vitamin B1, B2, B6, and B12 when compared with the control children.

## 4. Discussion

The major findings of the present study were: (1) children with ASD showed a DP characterized by relatively high consumption of cereals, pasta and dairy products, and a small intake of lean meat and eggs compared with the SENC guidelines [31]; (2) all children (control and ASD) consumed little fruit, vegetables, and fish. Instead, they ingested high amounts of fatty meat and its derivatives, as well as drinks, snacks, sweets, and baked goods confectionery. In particular, in children with ASD, the high intake of snacks, sweets, and bakery was associated with increased consumption of beverages and fat and lower consumption of fruits and vegetables; (3) the intake of meat of any type, both lean and fatty, was associated with higher consumption of fish and dietary fat. Also, the increased consumption of dairy products was associated with high consumption of cereals and pasta; (4) ASD children were grouped in a well-differentiated cluster from that of their control peers; (5) only about one-half of the children with ASD tolerated solid foods; (6) compared with control children, the percentages of ASD children complying with the adequacy of nutrient intakes were higher for energy, fat, saturated fat, calcium and vitamin C, and lower for fiber, iron, iodine, and vitamins of group B.

Families have related excessive consumption of cereals and dairy to an increased worsening of symptoms of ASD. However, systematic reviews on the gluten- and casein-free diet (GCFD) indicate that the evidence is insufficient to support or refute it [33,34,35,36]. Therefore, today GCFD for children with ASD cannot be recommended unless they are appropriately diagnosed with an allergy or intolerance to a certain compound or allergen. Instead, improvement of the DPs should be promoted by increasing consumption of fruits and vegetables, lean meat and fish, and decreasing consumption of enriched sugar and fatty food products, avoiding restricted diets unless there is a medical indication after a clear diagnosis of allergy or intolerance. In our study, even though having carried out a restriction diet for the treatment of ASD in the last 12 months was considered an exclusion criterion, none of the families interviewed reported using such diets.

Common DPs in children with ASD include a strong preference for processed foods, snacks, and starches coinciding with a bias against fruits and vegetables [37,38,39].

Some studies have shown that a DP with a low content of fibers (from legumes, nuts, and others) is associated with a reduced feeling of satiety during meals [40]. In addition, a higher intake of high energy density food, such as sweet cereals, sweets, and sugary drinks, constitutes an eating DP of high energy intake in all children. However, it is significant to note that this high-risk DP is more prominent among children with ASD, since their specificities in the consumption of some food groups lead them to a DP that diverges even further from the dietary guidelines (e.g., SENC guidelines) [21]. In the present work, except for dairy products, a relatively high proportion of children with ASD complied with the recommended food consumption guidelines of the SENC for pediatrics. In terms of food intake in ASD patients, there are some difficulties to consider which are not only related to their low tolerance of solid food but also with regard to their cognitive or behavioral rigidity, which determines a tendency in these patients to remain in a stable environment, and therefore makes the introduction of new foods difficult [16]. They also have difficulty in accepting certain textures, with alterations in palatability and also problems chewing and swallowing [41]. The intake of fruits and vegetables was lower than that recommended, mimicking what is usual in the whole Spanish population [42,43,44,45]. In agreement with our results, a study involving a sample of 70 children with ASD with severe food selectivity, i.e., complete omission of one or more food groups (e.g., fruit, vegetable, protein, grain, dairy) or consuming a narrow range of items weekly (e.g., five or fewer total food items) found that67% of the sample omitted vegetables and 27% omitted fruits [46].

One aspect of interest is the higher consumption of snacks, sweets, and baked goods in ASD patients compared with the SENC recommendations. However, the families reported that snacks were used as a positive reinforcement in psychoeducational therapies that these patients usually carry out. Besides that, these foods are sometimes used to introduce patients to solid foods in those with low tolerance. Even though the ingestion of snacks is not recommended except occasionally, we are aware of the importance of psychoeducational treatments based on selectivity towards some foods, including snacks [47]. Hence, although a flexible attitude about the ingestion of this type of food in ASD children is understandable, the high consumption of snacks, sweets, and baked goods in all Spanish children has been repeatedly reported [42,43,44,45], and should be modified.

Other authors have also reported alterations in eating behavior patterns in ASD. Compared to children with typical development, preschoolers with ASD consumed fewer vegetables, fish, and eggs, while primary school children consumed fewer legumes, cheese/yogurt, olive oil, citrus fruits, and more meat [21]. In terms of food tolerance, more than half of our children with ASD consumed a high percentage of pureed foods, particularly fruits, vegetables, fish, and fatty meats, despite being over two years old, which is when the bulk of the foods and modes of consumption of an adult have already been incorporated [48]. Indeed, the families mentioned they usually had jars of comminuted food, preferably chicken or beef with vegetables, and to a lesser extent fatty meats and fish. A recent study demonstrates that food selectivity and mealtime problems are common issues in preschoolers, school-age children, and adolescents with ASD, and they are associated with a higher frequency of gastrointestinal (GI) symptoms [49,50]. In our sample, at least in part, the increased consumption of shredded foods as well as the use of soft foods, e.g., porridges made by cereals with milk, and homogenized foods, might be due to the presence of minor GI symptoms.

Regarding nutrient intake, results in different studies are affected by many environmental and cultural factors. Sharp et al. (2013) [15] and Esteban-Figuerola et al. (2019) [51] have reported a lower intake of calcium and protein in autistic children compared with a control group, while here the intake of protein was higher than the recommendations and similar to the general population [15,51]. Likewise, the intake of fat for a majority of the ASD children complied with the recommendations, and even the intake of saturated fat was higher than in their control peers.

Essential fatty acids, LA and LNA, and their long-chain PUFA derivatives, mainly arachidonic acid (AA) and docosahexaenoic acid (DHA), play essential roles in growth and neurodevelopment, as well as in the prevention of diseases. Furthermore, low DHA levels have been associated with impaired language and motor skills in infants and children [52]. It has been suggested that children with ASD may be deficient in *n*-3 PUFA; in that sense, some studies have shown altered phospholipid–fatty acid compositions in plasma and red blood cells from children with ASD [53,54] and that dietary supplementation with EPA and DHA may contribute to improving the symptomatology [55,56,57,58,59]. However, the available data in this regard are scarce and often contradictory [34,59,60,61,62]. In contrast to other studies, in our sample the average daily intake of EPA + DHA in ASD children was about 0.125 g/d, close to the recommendations of the EFSA and other international entities e.g., FAO-OMS [63], and that value did not differ significantly from that observed in the control children. So these data do not seem to justify the use of omega 3 as a treatment in ASD, at least in Spain, where the familiar consumption of fish is traditionally higher compared to other countries [64]. In cases with low intake of fish, it could be recommended to plan a therapeutic test with supplementations to analyze blood levels fatty acids beforehand

Several studies have shown that children with ASD have inadequate micronutrient intakes [65,66], and they are at particular risk for specific inadequacies of vitamins and minerals such as calcium, magnesium, vitamin D, and vitamin E [39,46,51]. Children with ASD have also demonstrated low levels of folate, vitamins B6, and B12, which have been associated with simultaneous B6, B9, and B12 deficiencies leading to the accumulation of homocysteine [59,67]. In relation to the intake of calcium, our study shows different results compared with that of the Esteban-Figuerola et al. meta analysis (2019) [51]. In the present study, patients with ASD had a higher calcium intake than typically developing controls. This difference seems to be due to the increased consumption of dairy products reported by most of the families. This result can be contrasted with the tendency to use restriction diets in these patients, mainly GFCD, as described earlier, which implies the withdrawal of milk from the diet [33]. On the other hand, the limited intake of some micronutrients involved in the metabolism of 1-carbon fragments, such as folate, vitamin B12, and vitamin B6, may be critical because they contribute to the processes of DNA and histone methylation, which in turn influence the expression of numerous genes involved in neurodevelopment [68]. Moreover, these compounds prevent the accumulation of homocysteine in the brain, in addition to many other organs that are involved in increasing oxidative stress [69]. The etiology of ASD could be a relevant genetic component [70]. Nonetheless, environmental factors can contribute significantly to the disease’s evolution [71].

### Strengths and Limitations

Accurate quantification of dietary intake in free-living populations is a major challenge in nutritional epidemiology. Moreover, there is a strong debate over the validity of memory-based dietary assessment methods utilized in epidemiological research related to food group consumption and major events of disease [72]. In the present study, to evaluate the food consumption and estimate the nutrient intake, both a FFQ and a 24-hDR were used in a homogeneous sample of Spanish children from the same area and sharing the same socio-cultural habits. Because of its standardized format and the way these questionnaires are administered, they are methods with high performance in terms of cost-effectiveness, which has contributed to their widespread use in large epidemiological cohort studies and also with other designs. However, they have the disadvantage of incorporating systematic errors and biases, which is why procedures are currently being sought to improve the quality of the data collected [73].

This highlights that nutrient intakes variance is usually augmented due to day-to-day variation in individual intake, resulting in misleading estimates of low or high intakes [74]. To avoid the intra-individual variability of the data and obtain an estimate of the population’s usual intake distribution, repeated 24-h-DR must be used.

The use of validated tools for the assessment of DPs is limited. It has been suggested that the assessment of DPs, including their consistency and construct validity, should be evaluated over multiple administrations of the same dietary source, different dietary sources, or across various studies [75,76]. In the present study, we used both PCA and clustering analysis for the estimation of DPs. Based on the available evidence, most identified DPs showed good reproducibility, fair relative validity, and good construct validity across different statistical solutions [75].

One of the major limitations of our study is that we focused on a specific population of ASD children living in the southern of Spain. Therefore, our results should be cross-validated in other regions of Spain and other countries. Likewise, the sample of ASD children was very homogenous with regard to cultural and socio-economic aspects; thus, we cannot establish whether different eating patterns would affect nutrient intake adequacy. Finally, we did not attempt to evaluate gastrointestinal symptoms that could affect DPS and nutrient intakes.

## 5. Conclusions

In the present study, we reported differential DPs between children with ASD and children from the control using both PCA and hierarchical clustering analysis. This highlights high energy and fat consumption and frequently manufactured products with poor nutritional quality and a low intake of vegetables and fruits in ASD children. Likewise, this work adds further support to previous studies identifying inadequate micronutrient intakes for minerals like iron, iodine, and vitamins of group B. Adequate monitoring of the nutritional presence for these nutrients should be assessed and, if necessary, the use of supplements should be introduced into the diet. Hence, it seems relevant to assess the DPs and nutrient intakes in children with ASD to correct eating behavior disorders and rule out nutritional diseases.

## Figures and Tables

**Table 1:** Demographic and anthropometric characteristics of the group of children with autism spectrum disorders and the control healthy group.

Variables	Control (*n* = 57)	ASD (*n* = 54)	*p*-Value
Age (months)	51.5 (33–60)	42 (33–51)	0.07
Sex (male)	43 (75%)	45 (83.33%)	0.28
Weight (kg)	17.3 ± 2.5	16.7 ± 3.5	0.3
Height (cm)	106.3 ± 8.3	103.4 ± 9.7	0.6
BMI (kg/m^2^)	16.1 ± 1.7	15.9 ± 1.9	0.4

ASD: Autism Spectrum Disorders; BMI: Body Mass Index. The data have been expressed as mean ± standard deviation and absolute frequencies (%).

**Table 2:** Food group consumption according to food frequency questionnaires in Spanish preschool children with autism spectrum disorders (ASD).

Food Groups	Control (*n* = 57)	ASD (*n* = 54)
Cereals and pasta (s/d)	2.6 ± 1.0	4.6 ± 1.5 *
Fruits and vegetables (s/d)	2.0 ± 1.2	2.2 ± 0.9
Milk and dairy products (s/d)	3.2 ± 1.3	4.3 ± 1.6 *
Fish and shellfish (s/wk)	2.6 ± 1.1	2.5 ± 1.2
Lean meat and eggs (s/wk)	4.6 ± 1.4	2.0 ± 0.5 *
Fatty meats and derivatives (s/wk)	3.6 ± 2.3	2.8 ± 1.5
Fats (s/d)	2.8 ± 1.8	2.3 ± 0.6
Beverages (s/wk)	6.2 ± 3.1	4.1 ± 1.0 *
Snacks, sweets, bakery and pastry (s/wk)	5.5 ± 3.2	4.6 ± 2.8

Food servings are shown in mean ± standard deviation in servings per day (s/d) or servings per week (s/wk). Statistical differences were calculated using the U-Mann Whitney test between ASD and the control groups (* *p* < 0.05).

**Table 3:** Percentage of food group consumption (in servings per day or week) according to food frequency questionnaires and adequacy to the Spanish Society of Community Nutrition (SENC) dietary guidelines of preschool children with ASD compared with a healthy control group.

Food Groups (SENC Guidelines)	Control (*n* = 57)			ASD (*n* = 54)			*p*-Value
Cereals and pasta (4–6 s/d)	<4	4–6	>6	<4	4–6	>6	<0.001
	82.5	17.5	0	25.9	63	11.1	
Fruits and vegetables (≥5 s/d)	<3	>3		<3	>3		0.098
	87.7	12.3		96.3	3.7		
Milk and dairy products (2–4 s/d)	<2	2–4	>4	<2	2–4	>4	0.041
	10.5	71.9	17.5	3.7	59.3	37	
Fish and shellfish (3–4 s/wk)	>3	3–4	>4	>3	3–4	>4	0.22
	49.1	45.6	5.3	63	29.6	7.4	
Lean meat and eggs (3–5 s/wk)	<3	3–5	>5	<3	3–5	>5	<0.001
	7.0	61.4	31.6	94.4	5.6	0	
Fatty meats and derivatives (≤1 s/wk)	0	1	>2	0	1	>2	0.637
	1.8	10.5	87.7	1.9	16.7	81.5	
Fats (2–3 s/d) *	<2	2–3	>3	<2	2–3	>3	<0.001
	15.8	61.4	22.8	3.7	94.4	1.9	
Beverages (≤1 s/wk)	0	1	>2	0	1	>2	0.328
	0	1.8	98.2	0	0	100	
Snacks, sweets, bakery and pastry (≤1 s/wk)	0	1	>2	0	1	>2	0.511
	7	3.5	89.5	3.7	7.4	88.9	

SENC recommended food servings are shown in servings per day (s/d) or servings per week (s/wk), and the frequency of food consumption data are expressed as percentages (below). Statistical differences were calculated using a Chi-square test * Includes mostly olive oil for meal preparation.

**Table 4:** Adequacy of nutrient intake to the European Food Safe Authority (EFSA) recommendations of Spanish preschool children with autism spectrum disorders (ASD) compared with a healthy control group.

Variables	Control Group (*n* = 57)			Children ASD (*n* = 47)			*p*-Value
	Percentage below AI or RI	Percentage within RI	Percentage over AI or RI	Percentage below AI or RI	Percentage within RI	Percentage over AI or RI	
Energy	22.8		77.2	2.1		97.9	0.002
Proteins	0		100	0		100	1
Carbohydrates	14.3	76.8	8.9	21.3	76.6	2.1	0.254
Sugars	15.8		84.2	6.4		93.6	0.135
Fiber	50.9		49.1	34.0		66.6	0.085
Fats	50.9	28.1	21.1	23.4	38.3	38.3	0.014
Polyunsaturated fats	68.4		31.6	72.3		27.7	0.664
Saturated fats	47.4		52.6	23.4		76.6	0.012
Potassium	31.6		68.4	34.0		66.6	0.79
Calcium	49.1		50.1	21.3		78.7	0.003
Phosphorus	14.0		86	19.1		80.9	0.483
Magnesium	98.2		1.8	100		0	0.362
Iron	80.7		19.3	97.9		2.1	0.006
Zinc	70.2		29.8	80.9		19.1	0.211
Copper	96.5		3.5	100		0	0.195
Selenium	15.8		84.2	29.8		70.2	0.087
Manganese	100		0	100		0	1
Iodine	63.2		36.8	100		0	<0.001
Vitamin A	61.4		38.6	57.4		42.6	0.682
Vitamin D	91.2		8.8	95.7		4.3	0.36
Vitamin E	94.7		5.3	85.1		14.9	0.097
Vitamin K	57.9		42.1	44.7		55.3	0.179
Vitamin B1	56.1		43.9	91.5		8.5	<0.001
Vitamin B2	24.6		75.4	68.1		31.9	<0.001
Vitamin B3	96.5		3.5	100		0	0.195
Vitamin B6	33.3		66.7	53.2		46.8	0.041
Vitamin B9	91.2		8.8	91.5		8.5	0.962
Vitamin B12	24.6		75.4	48.9		51.1	0.01
Vitamin C	42.1		57.9	21.3		78.7	0.024

Nutrient adequacy was assessed by comparison of estimated usual individual intakes with the recommendations of adequate intake (AI) and reference intake range (RI) defined by EFSA [30]. Data are expressed as percentages of children below, within and over the recommended EFSA intake range for each nutrient. Percentage differences between ASD and control children were calculated using the Chi-square test.

## Data Availability

The datasets generated during and/or analyzed during the current study are available from the corresponding author upon reasonable request.
